# Phosphorylation of SMURF2 by ATM exerts a negative feedback control of DNA damage response

**DOI:** 10.1074/jbc.RA120.014179

**Published:** 2021-01-13

**Authors:** Liu-Ya Tang, Adam Thomas, Ming Zhou, Ying E. Zhang

**Affiliations:** 1 Laboratory of Cellular and Molecular Biology, Center for Cancer Research, National Cancer Institute, National Institutes of Health, Bethesda, Maryland, USA; 2 Protein Characterization Laboratory, Frederick National Laboratory for Cancer Research, Leidos Biomedical Research, Inc., Frederick, Maryland, USA

**Keywords:** ATM, SMURF2, RNF20, DNA damage response, H2B ubiquitination, ubiquitylation (ubiquitination), DNA repair, histone modification, phosphorylation

## Abstract

Timely repair of DNA double-strand breaks (DSBs) is essential to maintaining genomic integrity and preventing illnesses induced by genetic abnormalities. We previously demonstrated that the E3 ubiquitin ligase SMURF2 plays a critical tumor suppressing role via its interaction with RNF20 (ring finger protein 20) in shaping chromatin landscape and preserving genomic stability. However, the mechanism that mobilizes SMURF2 in response to DNA damage remains unclear. Using biochemical approaches and MS analysis, we show that upon the onset of the DNA-damage response, SMURF2 becomes phosphorylated at Ser^384^ by ataxia telangiectasia mutated (ATM) serine/threonine kinase, and this phosphorylation is required for its interaction with RNF20. We demonstrate that a SMURF2 mutant with an S384A substitution has reduced capacity to ubiquitinate RNF20 while promoting Smad3 ubiquitination unabatedly. More importantly, mouse embryonic fibroblasts expressing the SMURF2 S384A mutant show a weakened ability to sustain the DSB response compared with those expressing WT SMURF2 following etoposide treatment. These data indicate that SMURF2-mediated RNF20 ubiquitination and degradation controlled by ataxia telangiectasia mutated–induced phosphorylation at Ser^384^ constitutes a negative feedback loop that regulates DSB repair.

DNA double-strand breaks (DSBs) are the most deleterious type of DNA damage and need to be promptly and precisely repaired to preserve genomic integrity (1). Failure to do so can lead to cell death, sensitivity to genotoxic stresses, tissue degeneration, and cancer. In eukaryotes, DSBs are repaired through high-fidelity homologous recombination or error-prone nonhomologous end joining, depending on the cell cycle phase in which the DNA damage response is triggered (2, 3). Within minutes of DSB occurrence, a variant of histone 2A becomes phosphorylated at Ser^139^, forming γ-H2AX. This phosphorylation reaction is mediated by the ATM serine/threonine kinase, which is activated by DSB, and the rapid accumulation of γ-H2AX at DSBs initiates the DNA damage response through the recruitment of sensor proteins such as mediator of MDC1 (DNA damage checkpoint 1) (4) to the breaks, which in turn activates ATM in a positive feedback loop to enhance the γ-H2AX signal (5). The activated ATM also phosphorylates many other signaling molecules to realign transcription, translation, and cell cycle machinery toward DNA repair (6).

Eukaryotic DNA is wrapped around a core of eight histones and is further compacted into tightly organized chromatin, greatly inhibiting access to DNA by the repair machinery. As such, histone modification and subsequent chromatin decompaction plays a central role in regulating the DNA repair process (7). In addition to the phosphorylation of H2AX, ubiquitination, acetylation, and methylation of various histones play critical roles in reorganizing the chromatin structure and for recruiting and retaining DNA repair proteins at the sites of DSBs. This includes the monoubiquitination of histone H2B (ubH2B), which has a well-known role in opening chromatin in preparation for transcription (8, 9, 10). After DNA damage, H2B is ubiquitinated by a heterodimer of the RING finger E3 ligase, RNF20 (ring finger protein 20) and RNF40 (ring finger protein 40), which are orthologs of the budding yeast protein Bre1. RNF20–RNF40 and ubH2B were previously reported to be required for a timely DSB repair (11, 12).

SMURFs (Smad ubiquitin regulatory factors) are a subfamily of the HECT domain–containing E3 ligases. Since their initial discovery as negative regulators of TGF-β signaling, the repertoire of SMURF substrates has steadily expanded to include a large array of proteins involved in various cell functions (13). Previously, we showed that SMURF2 regulates the monoubiquitination of histone H2B by targeting RNF20 for polyubiquitination and proteasomal degradation, causing compaction of the chromatin structure (14). The profound impact of SMURF2 on the chromosomal landscape accounts for its important role as a tumor suppressor through maintaining genomic stability. However, SMURF2 and RNF20 are also rapidly recruited to the γ-H2AX foci upon the formation of DSBs, implying a role in the DNA damage response (14). Here we show that ATM phosphorylates SMURF2 at Ser^384^, and this phosphorylation is required for interaction between SMURF2 and RNF20, as well as polyubiquitination and degradation of RNF20. We further show that replacing WT Smurf2 in mouse embryonic fibroblasts (MEFs) with SMURF2 S384A renders the cells less sensitive to the cytotoxic effects of etoposide.

## Results

### Identification of a DNA damage–induced phosphorylation site of SMURF2 at Ser^384^

**Figure 1:**
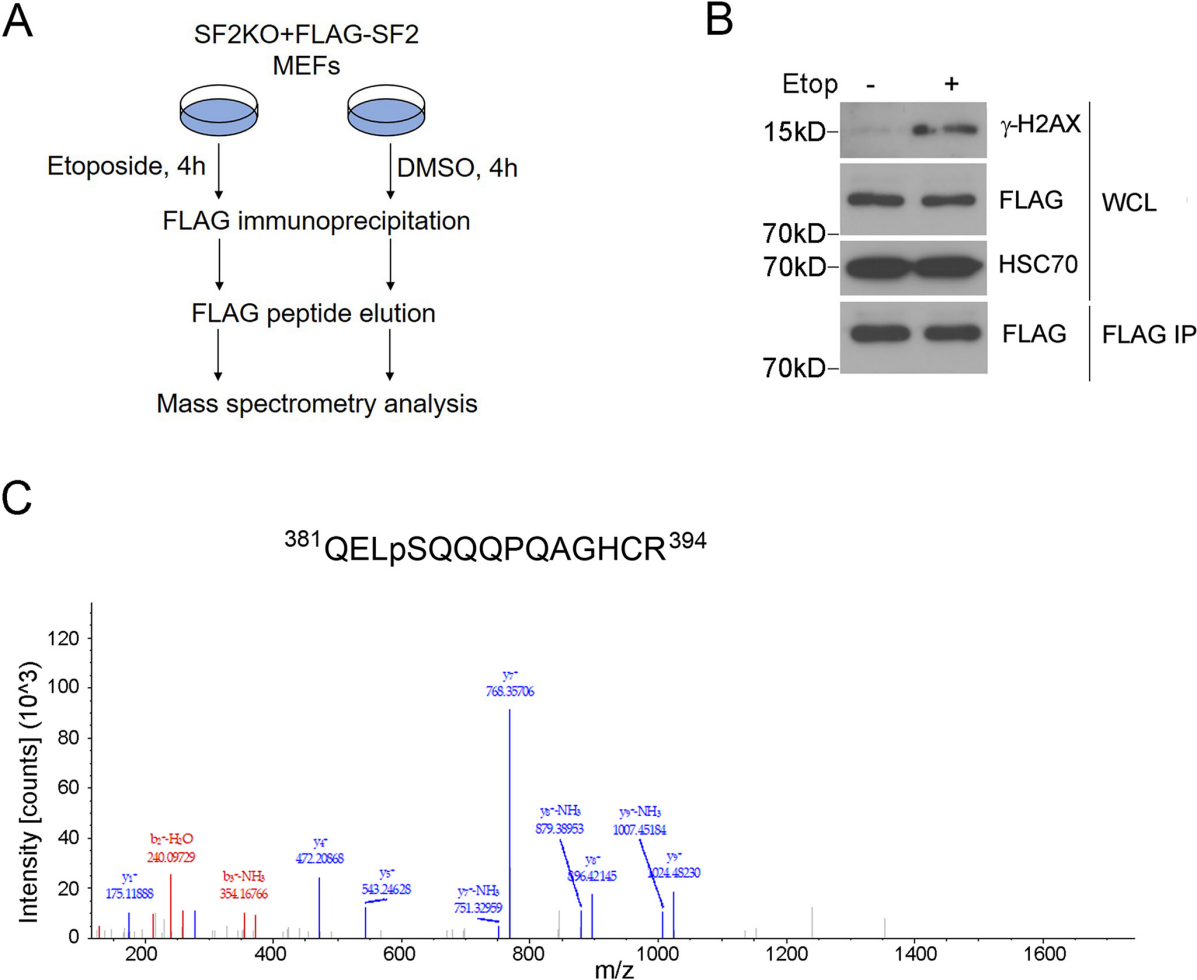
**SMURF2 Ser^384^ phosphorylation is identified in etoposide-treated MEFs.***A*, the experimental workflow. *Smurf2*^−/−^ MEFs, which were stably expressing FLAG–SMURF2, were treated with 50 μm etoposide (*Etop*) or DMSO for 4 h. The cell lysates were subjected to FLAG IP and FLAG peptide elution. MS was applied to identify the phosphorylation site(s) on the eluted SMURF2 protein. *B*, Western blotting analysis of whole cell lysates (*WCL*) and FLAG peptide elution fraction. *C*, the mass spectrum of the phosphorylation peptide that harbors the Ser^384^ phosphorylation.

To determine the molecular mechanisms that regulate SMURF2 activity during the DNA damage response, we undertook an approach to identify post-translational modifications of SMURF2 in response to etoposide, a topoisomerase II inhibitor that induces DSBs (Fig. 1*A*). Etoposide treatment of *Smurf2*^−/−^ MEFs that were reconstituted with stably expressed FLAG–SMURF2 induced γ-H2AX (Fig. 1*B*). FLAG–SMURF2 was then purified from these cells using anti–FLAG–agarose affinity beads followed by elution with free FLAG peptide (Fig. 1*B*). MS analysis of the treated and untreated eluates identified several modifications on SMURF2 (Tables S1 and S2), including a differentially phosphorylated site at Ser^384^ of SMURF2 that was only detected in etoposide-treated cells (Fig. 1*C*). Because the amino acid sequence surrounding Ser^384^ closely resembles the SQ motif of the ATM kinase recognition sequence (15) (Fig. 1*C*), our result suggested that SMURF2 could be regulated by ATM upon formation of DSBs.

### ATM physically interacts and phosphorylates SMURF2

**Figure 2:**
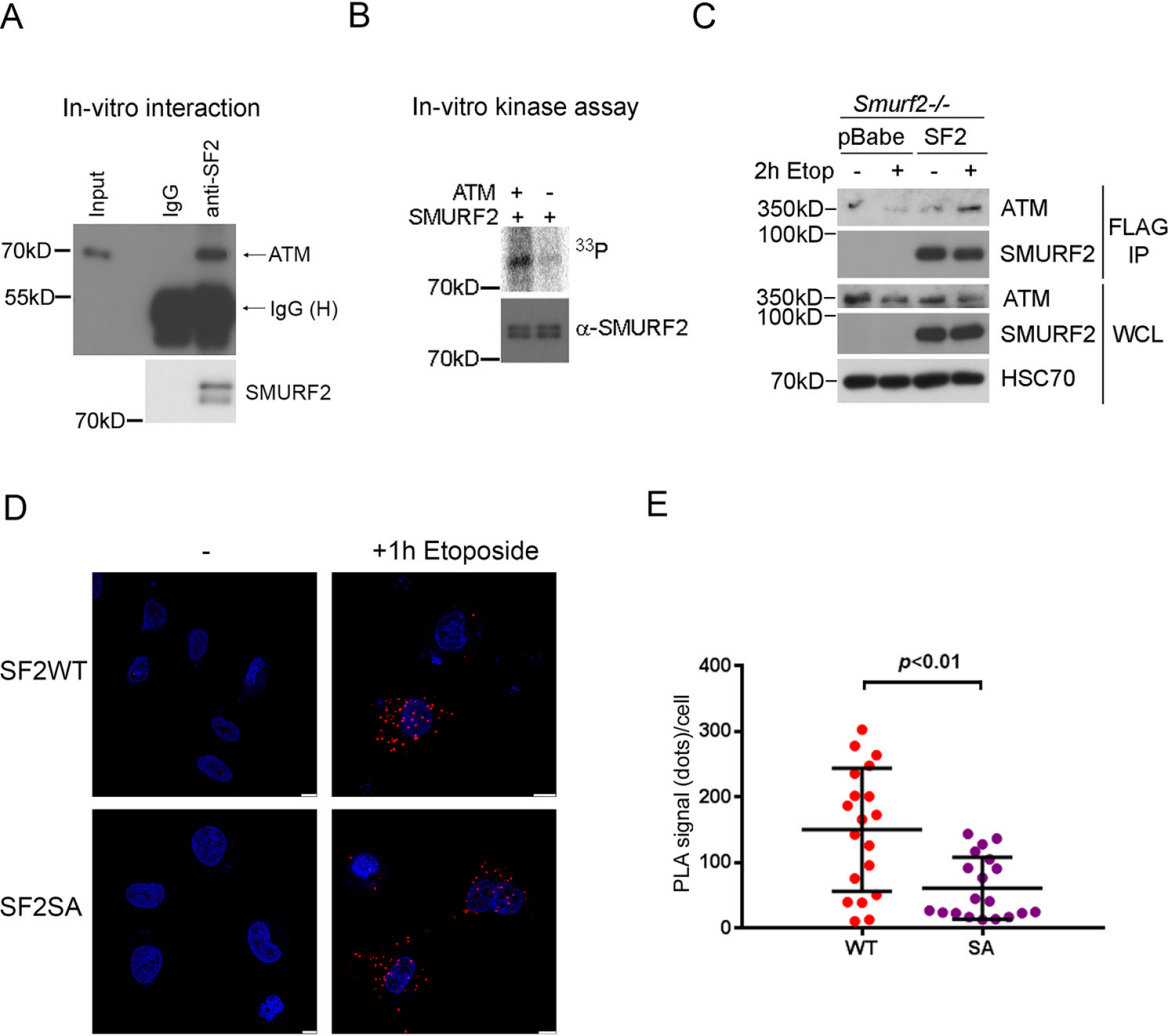
**ATM directly interacts with and phosphorylates SMURF2, and the interaction between ATM and SMURF2 was induced by etoposide treatment.***A*, ATM directly interacts with SMURF2. *B*, ATM directly phosphorylates SMURF2. *In vitro* kinase assay was performed by incubating recombinant His_6_–SMURF2 and ATM in the presence of [^33^P]ATP. *C*, SMURF2 interacts with ATM upon etoposide (*Etop*) treatment. *Smurf2*^−/−^ MEFs, which were stably expressing control vector pBabe or FLAG–SMURF2, were treated with DMSO or etoposide for 2 h. The cell lysates were subjected to FLAG immunoprecipitation and Western blotting analysis. *WCL*, whole cell lysate. *D*, SMURF2 and ATM interacts in U2OS cells. U2OS cells, which transiently expressed FLAG–SMURF2 (WT or SA mutant), were treated with etoposide for 1 h. The cell sample was analyzed by PLA using primary antibodies that recognize FLAG or ATM, respectively. *Scale bar*, 10 μm. *E*, quantitation of PLA signals showed that interaction between SMURF2 (SA) and ATM was weaker than that of SMURF2 (WT).

ATM is known for its role as the chief mobilizer and activator of the DNA damage response in response to DSBs by phosphorylating many downstream effectors (6). To test whether ATM directly interacts with SMURF2, we mixed purified recombinant His_6_–SMURF2 and GST–ATM proteins and performed immunoprecipitation. The results showed specific presence of ATM proteins in the anti-SMURF2 immunoprecipitates (Fig. 2*A*), indicating that SMURF2 and ATM can directly bind to each other. We also incubated recombinant His_6_–SMURF2 and active ATM proteins in the presence of [^33^P]ATP and found that SMURF2 was phosphorylated by ATM (Fig. 2*B*). This result strongly suggests that SMURF2 is a direct substrate of ATM-mediated phosphorylation. Immunoprecipitation (IP) of FLAG–SMURF2 expressed in *Smurf2*^−/−^ MEFs also identified endogenous ATM in the immunoprecipitates, especially after etoposide treatment (Fig. 2*C*). We further verified the interaction between SMURF2 and ATM by proximity ligation assay (PLA) after expressing FLAG–SMURF2 in U2OS cells. Upon initiation of DSB and ATM activation by etoposide treatment, distinct speckles representing co-localized FLAG–SMURF2 and ATM were detected in the nucleus of treated but not untreated cells (Fig. 2*D*). Finally, to address the role of phosphorylation at Ser^384^, we made a phosphorylation-resistant Ser^384^-to-Ala mutant, SMURF2 (SA), and repeated the PLA experiment as described above. The result showed a significant reduction in the number of PLA speckles between ATM and the SA mutant SMURF2 (Fig. 2, *D* and *E*). Similar results were obtained by PLA in U2OS cells after treating cells with camptothecin, a topoisomerase poison that also induces DNA damage response (Fig. S1).

### Phosphorylation at Ser^384^ is required for SMURF2-mediated RNF20 ubiquitination

**Figure 3:**
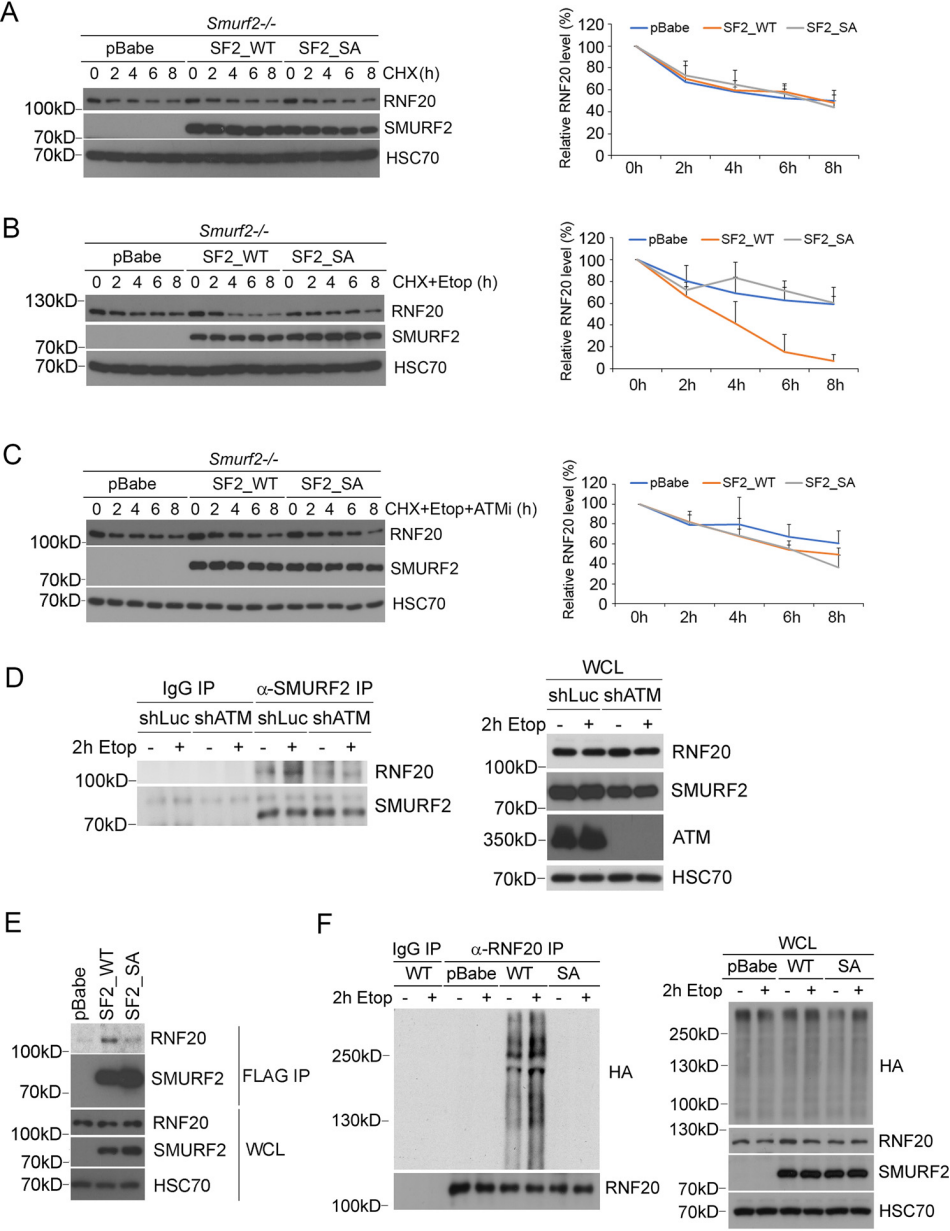
**SMURF2 Ser^384^ phosphorylation affects the SMURF2-induced RNF20 ubiquitination.***A*, the RNF20 protein levels were similar among *Smurf2*^−/−^ MEFs stably expressing control vector pBabe, SMURF2 (WT), or SA upon cycloheximide treatment. *B*, upon both cycloheximide and etoposide treatment, the RNF20 protein level was decreased in *Smurf2*^−/−^ cells expressing SMURF2 (WT), compared with the RNF20 protein level in the cells expressing control vector or SMURF2 (SA). *C*, ATM inhibitor restored the RNF20 protein level in *Smurf2*^−/−^ cells expressing SMURF2 (WT) in the presence of cycloheximide and etoposide treatment. Quantitation of RNF20 protein levels from three independent experiments is shown in the *right panels*. *D*, the interaction between RNF20 and SMURF2 is regulated by ATM and etoposide (*Etop*) treatment. Control (shLuc) or ATM knockdown (shATM) USOS cells were treated with DMSO or etoposide and then subjected to SMURF2 IP. The presence of RNF20 was examined by Western blotting. *WCL*, whole cell lysate. *E*, RNF20 preferentially interacts with SMURF2 (WT). *Smurf2*^−/−^ MEFs, which were stably expressing FLAG–SMURF2 (WT or SA) or pBabe vector, were subjected to FLAG IP and Western blotting analysis. *F*, SMURF2 (WT) but not SA is required for the polyubiquitination of RNF20. HA–ubiquitin was transfected to *Smurf2*^−/−^ MEFs expressing SMURF2 (WT) or SA mutant, and the cells were treated with MG132 with or without etoposide. After RNF20 IP, the ubiquitination signal was visualized by Western blotting.

During the DNA-damage response, SMURF2 is recruited to DSBs where it interacts with and induces polyubiquitination of RNF20, leading to RNF20 degradation (14). To determine whether this process is subject to control by the ATM-mediated phosphorylation of SMURF2 at Ser^384^, we generated a pair of stable *Smurf2*^−/−^ MEF cell lines expressing SMURF2 (WT), or SMURF2 (SA) mutant, and compared the stability of RNF20 in these two cell lines by Western blotting in the presence of cycloheximide to block protein synthesis. Under normal culturing conditions, the half-lives of RNF20 in *Smurf2*^−/−^ MEFs expressing SMURF2 (WT) or SMURF2 (SA) were quite comparable (Fig. 3*A*). However, upon etoposide treatment, the turnover rate of RNF20 in the SMURF2 (WT)–expressing cells was much faster than that in the vector control or SMURF2 (SA)–expressing cells (Fig. 3*B*). Moreover, adding the ATM inhibitor KU60019 blocked faster turnover of RNF20 in the presence of etoposide (Fig. 3*C*). These results suggest that the ATM activity is essential for the etoposide-induced RNF20 turnover, and SMURF2 E3 ligase activity was likely compromised by the SA mutation. To determine whether this was the case, we isolated SMURF2 by IP in U2OS cells expressing either control shLuc or shATM vectors and found that SMURF2 only interacted with RNF20 in the presence of ATM upon etoposide treatment (Fig. 3*D*), indicating that ATM is required for the SMURF2 and RNF20 interaction. Furthermore, in *Smurf2*^−/−^ MEFs expressing SMURF2 (WT) or SA mutant, we found that SMURF2 (SA) bound much less RNF20 than SMURF2 (WT) (Fig. 3*E*), indicating a reduced affinity of SMURF2 (SA) toward RNF20. We also introduced HA–ubiquitin into *Smurf2*^−/−^ MEFs expressing SMURF2 (WT) or SA mutant and treated the cells with MG132, which blocks proteasome-mediated protein degradation, and analyzed the level of RNF20 ubiquitination in the absence or presence of etoposide. The results showed that etoposide treatment enhanced polyubiquitination of RNF20, and the ubiquitination of RNF20 requires SMURF2 (WT) but not SMURF2 (SA) (Fig. 3*F*).

**Figure 4:**
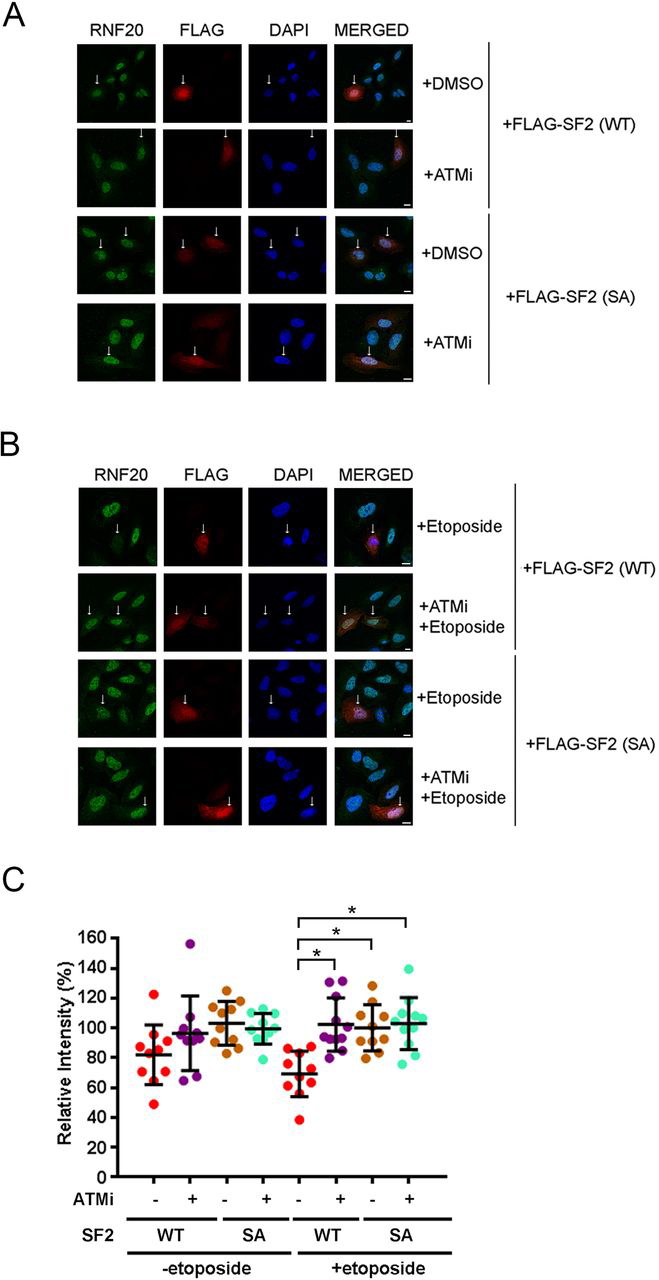
**ATM activity is required for SMURF2-induced down-regulation of RNF20 protein.***A*, FLAG–SMURF was transiently expressed in U2OS cells. Without etoposide treatment, the RNF20 protein level remained similar in transfected cells (indicated by *arrows*) and nontransfected neighbor cells. *DAPI*, 4′,6-diamino-2-phenylindole. *B*, upon etoposide treatment, the presence of SMURF2 (WT) decreased the RNF20 protein level, whereas SMURF2 (SA) cannot. The effect of SMURF2 (WT) on RNF20 protein level was blocked by the ATM inhibitor. *Scale bar*, 10 μm. *C*, the relative fluorescence intensity of RNF20 was quantitated in the cells expressing SMURF2 (WT) or SA with the indicated treatments. Statistically significant differences (*p* < 0.01) are indicated by *asterisks*.

The regulation of SMURF2-mediated RNF20 down-regulation during the DNA-damage response can be directly visualized in individual cells by immunofluorescence (14). To assess the impact of ATM-mediated phosphorylation of SMURF2 in this setting, we transfected FLAG–SMURF2 (WT) or FLAG–SMURF2 (SA) into U2OS cells. In the absence of etoposide treatment, RNF20 protein level remained similar between FLAG–SMURF2 transfected cells and nontransfected neighboring cells regardless of whether the cells were treated with the ATM inhibitor (Fig. 4*A*). In contrast, after activation of the DNA-damage response by etoposide, RNF20 fluorescence (protein level) disappeared wherever the cells were positively transfected with FLAG–SMURF2 (WT). Importantly, blocking ATM activity restored RNF20 protein levels (Fig. 4*B*). On the other hand, RNF20 fluorescence still persisted in FLAG–SMURF2 (SA) transfected cells, whether or not ATM was inhibited (Fig. 4*B*). The relative fluorescence intensity of RNF20 proteins in transfected cells compared with that in nontransfected cells and statistical analyses is presented in Fig. 4*C*. Taken together, the above results indicate that ATM-mediated SMURF2 Ser^384^ phosphorylation is required for SMURF2-mediated RNF20 ubiquitination and degradation.

### Role of Ser^384^ phosphorylation is likely specific to SMURF2 regulation of RNF20

**Figure 5:**
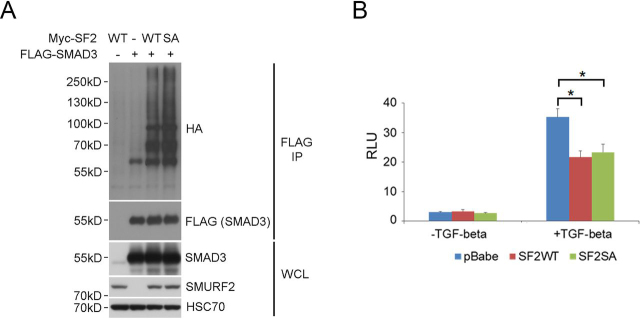
**The Ser^384^ phosphorylation has no effect on SMURF2's function in TGF-β signaling.***A*, SMAD3 ubiquitination is not affected by SMURF2. HA–ubiquitin, FLAG-SMAD3, and Myc-SMURF2 (WT or SA) were transfected to *Smurf2*^−/−^ MEFs. After FLAG IP, the ubiquitination signal was visualized by Western blotting. *WCL*, whole cell lysate. *B*, the inhibitory effect of SMURF2 on TGF-β signaling is not affected by SA mutation. FLAG–SMURF2 (WT or SA), Smad-responsive luciferase reporter (CAGA_12_-Luc), and pTK–*Renilla*–*Luciferase* plasmid were transfected into *Smurf2*^−/−^ MEFs. 24 h after transfection, the cells were treated with TGF-β for 20 h. The firefly luciferase activities were normalized to the *Renilla* luciferase activities. An *asterisk* indicates statistically significant differences (*p* < 0.01) compared with pBabe vector control cells upon TGF-β stimulation.

SMURF2 plays important roles in a diverse array of cellular functions including promoting SMAD3 monoubiquitination, which inhibits TGF-β–induced SMAD-dependent transcriptional responses (16). To investigate whether phosphorylation at Ser^384^ influences other aspects of SMURF2 function or exerts a unique control of RNF20, we first compared levels of SMAD3 ubiquitination after transiently transfecting HA–ubiquitin, FLAG–SMAD3, and Myc–SMURF2 (WT) or Myc–SMURF2 (SA) in *Smurf2*^−/−^ MEFs. The results showed comparable levels of SMAD3 ubiquitination (Fig. 5*A*). Then, using a SMAD3-dependent (CAGA)_12_-Luc reporter, we found that both SMURF2 (WT) and SA mutant were capable of suppressing the TGF-β–induced transcriptional response (Fig. 5*B*). These results indicate that phosphorylation at Ser^384^ of SMURF2 is likely a specific form of control of SMURF2 function in the DNA-damage response, whereas it has no impact on TGF-β signaling *per se*.

### SMURF2 Ser^384^ phosphorylation delays timely DSB repair

**Figure 6:**
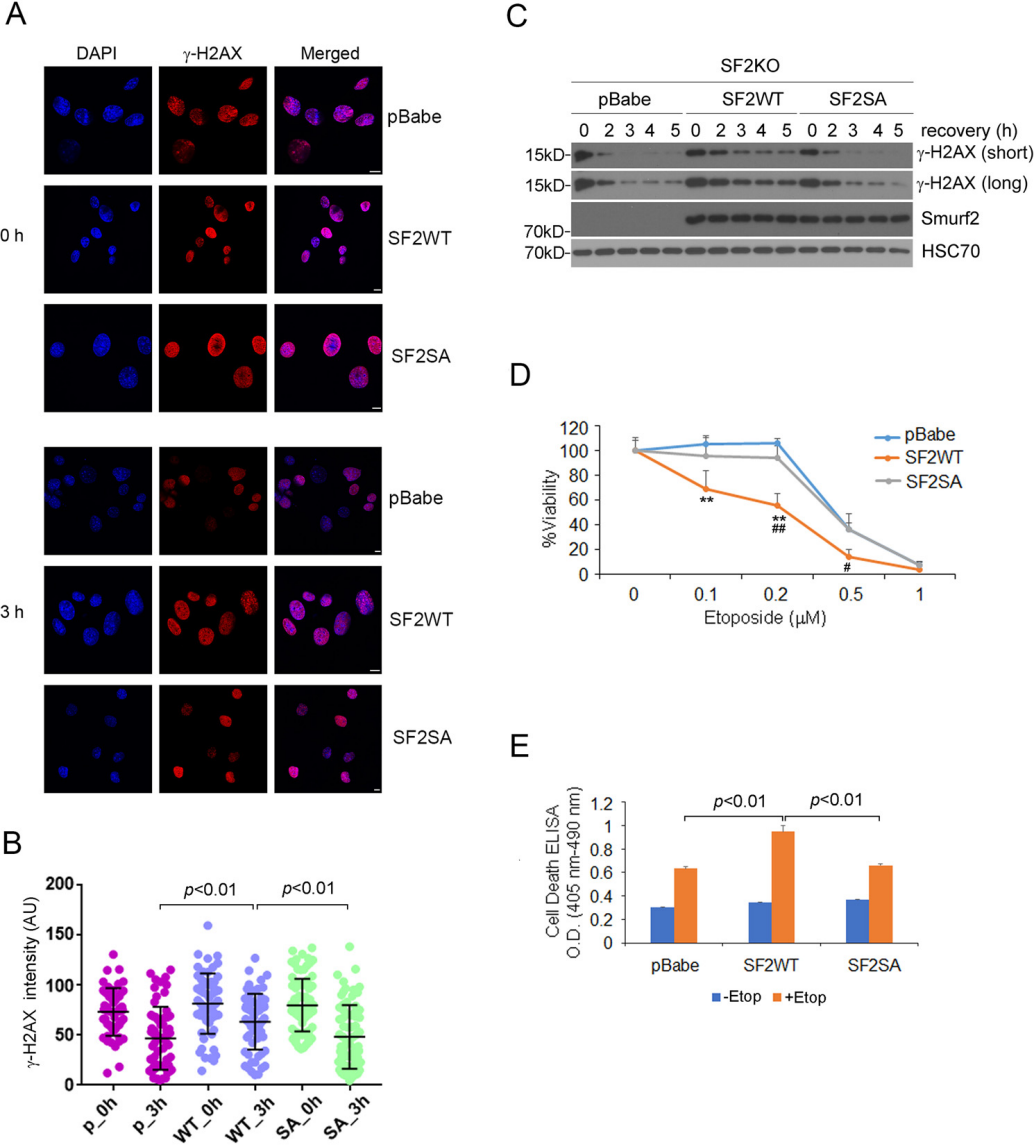
**SMURF2 Ser^384^ phosphorylation affects the clearance of** γ**-H2AX in the DSB repairing process and sensitivity of cells in response to DNA damage.***A*, immunofluorescence staining shows the higher γ-H2AX level in *Smurf2*^−/−^ stable cells expressing SMURF2 (WT) after etoposide treatment. *Smurf2*^−/−^ MEFs, which were stably expressing SMURF2 (WT) or SA mutant or control vector, were treated with etoposide for 2 h and then recovered for 3 h before immunostaining. *DAPI*, 4′,6-diamino-2-phenylindole. *Scale bar*, 10 μm. *B*, the quantitation result of γ-H2AX level in Fig. 6*A*. *C*, Western blotting shows accumulated γ-H2AX level in *Smurf2*^−/−^ stable cells expressing SMURF2 (WT) after etoposide treatment. *Smurf2*^−/−^ MEFs, which were stably expressing SMURF2 (WT) or SA mutant or control vector, were treated with etoposide for 2 h and then recovered for the times indicated. *D*, the cell viability of *Smurf2*^−/−^ stable cells after etoposide treatment. *Smurf2*^−/−^ MEFs, which were stably expressing SMURF2 (WT) or SA mutant or control vector, were treated with indicated concentrations of etoposide for 60 h. The cell viability assay was done in IncuCyte, and the cell viability was calculated by cell growth area relative to the untreated control. *Double asterisks* indicate statistically significant differences (*p* < 0.01) compared with pBabe vector control cells. *Single* or *double hashtags* indicate statistically significant differences (*p* < 0.05 or *p* < 0.01, respectively) compared with SMURF2 (SA) cells. *E*, assessment of the rate of cell death induction in *Smurf2*^−/−^ stable cells (pBabe, SMURF2 (WT), or SMURF2 (SA)) upon etoposide (*Etop*) treatment.

When mobilized, RNF20 is known to heterodimerize with RNF40 to induce ubH2B, which is required for the timely repair of DSBs (11). Thus, the fact of promoting RNF20 degradation via the ubiquitin-proteasome system places SMURF2 as a negative regulator in the DNA damage response. To determine whether SMURF2 Ser^384^ phosphorylation plays a regulatory role in DSB repair, we measured the rate of γ-H2AX disappearance following etoposide withdrawal, a surrogate marker of DSB repair. Treating *Smurf2*^−/−^ MEFs with etoposide for 2 h led to robust accumulation of γ-H2AX at DNA damage sites regardless of whether the cells expressed WT SMURF2 or the mutant (Fig. 6*A*). Following etoposide withdrawal from the culture medium, γ-H2AX rapidly disappeared within 3 h in *Smurf2*^−/−^ MEFs expressing either the vector control or SMURF2 (SA), but its level still persisted in cells expressing SMURF2 (WT) (Fig. 6*A*). These changes in γ-H2AX accumulation at DNA damage sites were confirmed by direct visualization using immunofluorescence and Western blotting (Fig. 6, *A–C*). These results were consistent with previously published results on RNF20 knockdown (11), suggesting that Ser^384^ phosphorylation of SMURF2 is functionally equivalent to RNF20 removal for the control of DSB repair.

The abrogation of the DNA damage repair process is often associated with increased sensitivity of cells to the cytotoxic effect of the drugs that induce DNA damage (17). Indeed, we observed that *Smurf2*^−/−^ MEFs reconstituted with SMURF2 (WT) were much more sensitive to etoposide compared with those reconstituted with control vector or the SA mutant (Fig. 6*D*). In addition, more cell death was observed in the former than the latter two groups of cells (Fig. 6*E*). These results indicate that delayed DSB repair rendered cells more sensitive to etoposide and suggest that the DNA damage response induced by the RNF20-ubH2B axis is regulated by ATM-mediated phosphorylation of SMURF2 at Ser^384^.

## Discussion

ATM is a primary transducer of the DSB response by phosphorylating a plethora of effectors in various DNA damage response pathways (6). Upon DNA damage, a fraction of RNF20 and RNF40 is recruited to DSB sites and undergoes ATM-mediated phosphorylation (11). The ATM-mediated phosphorylation of RNF20 and RNF40 are required for DNA damage-induced monoubiquitination of H2B, which is essential to timely DSB repair (11). In this study, we showed that ATM can also phosphorylate SMURF2, and this phosphorylation is required for the ability of SMURF2 to interact, ubiquitinate, and degrade RNF20. Consistent with the positive role of RNF20 in DSB repair, our data suggest that ATM-mediated SMURF2 phosphorylation acts as a negative feedback control by reducing RNF20 levels.

In contrast to their well-characterized functions in transcription, the mechanisms of RNF20, RNF40, and ubH2B actions in DNA damage response are still elusive. It is known that monoubiquitination of H2B mediated by RNF20 and RNF40 relaxes the chromatin (10). We previously showed that loss of SMURF2 results in the up-regulation of RNF20 and ubH2B, which in turn relaxes the chromatin, making the DNA more accessible to DNA damaging agents as evidenced by increased γ-H2AX foci upon challenge with etoposide (14). On the other hand, decondensed chromatin resulting from increased ubH2B modification allows a subset of repair proteins to access the DNA to promote repair (11). Our current study indicates that the RNF20 level is regulated by ATM-mediated phosphorylation of SMURF2. In the absence of this phosphorylation or absence of SMURF2, DSB repair occurs much faster, as shown by the rapid disappearance of γ-H2AX at DSB. This suggests that by regulating RNF20, SMURF2 plays dual roles in DNA damage response: SMURF2-mediated RNF20 ubiquitination down-regulates ubH2B, thereby promoting chromatin compaction and protecting cells from DNA damage insult, while at the same time, it could interfere with the recruitment of DNA repair proteins to DSB sites for timely repair.

Phosphorylation is known to influence the catalytic activity of the HECT domain–containing E3 ligases. Previously, phosphorylation of NEDD4.1 and ITCH was shown to cause conformation changes by relieving their autoinhibition fold (18, 19). However, very little is known about the phosphorylation control of SMURF2 activity. Our current study provides an example of regulating substrate specificity by phosphorylation of SMURF2. Because ATM is only activated upon DNA damage, it is expected that SMURF2 could be phosphorylated by other kinases. Indeed, a recent study found that SMURF2 can be phosphorylated by the mitogen-activated protein kinase Erk5 at Thr^249^, which promotes proteasomal degradation of Smad1 during mammalian skeletogenesis (20). More detailed characterization of various post-translational modifications on SMURF proteins will be a rich area for future investigation.

## Experimental procedures

### Antibodies and reagents

Antibodies to FLAG (catalog no. F1804) and ATM (catalog no. PLA0086) were purchased from Sigma. Antibodies to phospho-histone-H2AX (Ser^139^) (catalog no. 9718), and DYKDDDDK (equivalent to FLAG, catalog no. 8146) were purchased from Cell Signaling Technology. Antibodies to RNF20 (catalog no. ab181104), SMURF2 (catalog no. ab53316), and GST (catalog no. ab19256) were obtained from Abcam. Anti-HSC70 (catalog no. sc-7298) was purchased from Santa Cruz. Cycloheximide was purchased from Merck Bioscience. Etoposide and KU60019 (ATM inhibitor) were purchased from Sigma. Purified GST–ATM protein (catalog no. A26-35G) for the *in vitro* binding assay was obtained from SignalChem, and purified active ATM for *in vitro* kinase assay was purchased from Sigma (catalog no. 14-933M). Agarose-conjugated anti-FLAG (catalog no. A2220), FLAG peptide (catalog no. F3290), and anti–HA-peroxidase (catalog no. 12013819001) were obtained from Sigma. All other antibodies and reagents used in this study have been described previously (14).

### Expression plasmids and transfection

HA-tagged ubiquitin, FLAG–SMAD3, Myc–Smurf2, pBabe–FLAG–Smurf2–puro, (CAGA)_12_-Luc, and pTK–*Renilla*–*Luciferase* vectors were described previously (14, 16). SMURF2 S384A mutation was generated using a PCR-based strategy using the primers 5′-GAGGCTGTTGTTGGGCAAGTTCTTGCCGCAAA-ATTTT-3′ (forward primer) and 5′-AAAATTTTGCGGCAAGAACTTGCCCAACAACAGCCTC-3′ (reverse primer) and subcloned into pBabe–FLAG–puro or pRK–Myc vector. The mutation site was verified by sequencing. All the transfection experiments were performed using Lipofectamine 3000 (Thermo) according to the manufacturer's protocol.

### Cell culture

Human U2OS cells were obtained from the American Type Culture Collection. The establishment of immortalized *Smurf2*^−/−^ MEFs was described previously (16). shLuc and shATM U2OS cells were provided by Dr. Y. Shiloh (21). MEFs and U2OS cells were cultured in Dulbecco's modified Eagle's medium supplemented with 10% fetal bovine serum. For the reconstitution of SMURF2 (WT) or SMURF2 (SA) in *Smurf2*^−/−^ cells, immortalized MEFs were infected with retroviral particles containing pBabe–FLAG–Smurf2–puro or Smurf2 (SA) vector.

### FLAG immunoprecipitation and mass spectrometric analysis

MEF stable cells expressing control vector (pBabe) or SMURF2 were treated with 50 μm etoposide for 4 h. The cell lysates from two cell groups were subjected to FLAG IP, and the protein complex was eluted by 1 mg/ml FLAG peptide. The FLAG peptide elution was digested with trypsin according to the protocol described previously (22).

For mass spectrometric analysis, an aliquot (6 μl) of each sample was loaded on an Easy nLC II nano-capillary HPLC system (Thermo Scientific) with a C18 Nano trap column, (2 cm, nanoViper, Thermo Scientific) and a C18 Nano analytical column (15 cm, nanoViper, Thermo Scientific) coupled online with an Q Exactive^TM^ HF Hybrid Quadrupole-Orbitrap^TM^ mass spectrometer (Thermo Scientific). A linear gradient of 2% mobile phase B (acetonitrile with 0.1% formic acid) to 42% mobile phase B within 45 min at a constant flow rate of 200 nl/min was used to elute the peptides. The 12 most intense molecular ions in each MS scan were sequentially selected for high-energy collision dissociation using a normalized collision energy of 29%. The mass spectra were acquired at the mass range of *m*/*z* 350–2000. Nanospray Flex^TM^ ion sources (Thermo Scientific) capillary voltage and temperature were set at 1.7 kV and 300 °C, respectively. The radio frequency (RF) lens was set at 60%. The dynamic exclusion function on the mass spectrometer was enabled during the MS2 data acquisition. The MS data were first searched against a combined database containing human SMURF2 (SwissProt no. Q9HAU4) and *Mus musculus* fasta database/SwissProt/TrEMBL (released in January 2016, 47,929 entries) utilizing SEQUEST HT interfaced with Proteome Discoverer 1.4 (Thermo Scientific) and then again with a combined database containing human SMURF2 (SwissProt no. Q9HAU4) and *M. musculus* subset of the SwissProt database (released in August 2020, 17,023 entries) with Proteome Discoverer 2.4 (Thermo Scientific). Up to two missed tryptic cleavage sites were allowed. The oxidation of methionyl residue and phosphorylation on serine, threonine, and tyrosine were included as a dynamic modification. The precursor ion tolerance was set at 20 ppm, and the fragment ion tolerance was set at 0.02 Da. The peptide identifications were filtered through protein Percolator with the cutoff of a false peptide discover rate less than 1% for all peptides identified.

### In vitro binding assay

GST–ATM (0.25 µg) was incubated with His_6_–SMURF2 (0.4 µg) for 2 h at 4 °C, and protein A/G–agarose together with anti-SMURF2 antibody or rabbit IgG was added into the protein complex. After overnight incubation at 4 °C, the supernatant was removed, and the agarose was thoroughly washed. The protein complex was eluted with 2× SDS-PAGE protein sample buffer (80 mm Tris-HCl, 2% SDS, 10% glycerol, 100 mm DTT, 0.0006% bromphenol blue) and subjected to Western blotting analysis.

### In vitro kinase assay

His_6_–SMURF2 (0.88 µg) was incubated with or without active ATM (0.38 µg) in the presence of 5 μCi of [γ-^33^P]ATP (3,000 μCi/mmol) in 1× kinase buffer (10 mm Hepes-KOH, pH 7.5, 5 mm MgCl_2_, 5 mm MnCl2, and 5 mm CaCl_2_) at 37 °C. After a 30-min incubation, the reaction was stopped by adding an equal volume of 2× SDS-PAGE protein sample buffer and subjected to autoradiography.

### Immunofluorescence and PLA

For measuring RNF20 protein level after etoposide and/or ATM inhibitor treatment, U2OS cells, which transiently expressed FLAG–SMURF2 (WT or SA mutant), were treated with the ATM inhibitor KU60019 or DMSO for 2 h and then treated with 50 μm etoposide or DMSO for 1 h. After treatment, the cells were stained with anti-FLAG (monoclonal) and anti-RNF20 (polyclonal). To test the γ-H2AX protein level in DNA damage repair, *Smurf2*^−/−^ stable MEFs (pBabe, SMURF2 (WT), or SMURF2 (SA)) were treated with etoposide for 2 h and then recovered for 3 h. All immunofluorescence images were captured by using a Leica TCS SP8 confocal system and analyzed by Imaris 8 (Oxford Instruments).

The PLA was performed using the Duolink® system (Sigma–Aldrich) according to the manufacturer's instructions. Briefly, U2OS cells were grown on BD Falcon four-chamber slides and transiently transfected with FLAG–SMURF2 (WT or SA mutant). The cells were treated with 50 μm etoposide for 1 h and proceeded to PLA protocol. Anti-FLAG (monoclonal) and anti-ATM (polyclonal) primary antibodies were added together to the cell sample and incubated at 4 °C overnight. After washing steps, the cell sample was sequentially incubated with secondary antibodies with PLA probes, ligation solution, and detection solution with thorough washes between each step. PLA signals were visualized using a Leica TCS SP8 confocal system. Statistical analyses of PLA data were performed using BlobFinder (24).

### Cell viability and cell death assay

For the cell viability assay, *Smurf2*^−/−^ stable MEFs (expressing pBabe, SMURF2 (WT), or SMURF2 (SA)) were seeded at 2 × 10^3^ cells/well in 96-well plate and treated with etoposide with a concentration from 0 to 1 μm. The plate was inserted into the Incucyte ZOOM for real-time imaging, with four fields imaged per well every 4 h for a total of 60 h. The cell viability was calculated based on the relative ratio of cell growth area. To measure cell death, *Smurf2*^−/−^ stable MEFs (pBabe, SMURF2 (WT), or SMURF2 (SA)) were plated at 2 × 10^5^ cells/well in 6-well plates and treated with 20 μm etoposide or DMSO for 48 h, and the cell death was analyzed by using a cell death detection ELISA kit (Roche), a photometric enzyme immunoassay for the qualitative and quantitative *in vitro* determination of cytoplasmic histone-associated DNA fragments (mono and oligonucleosomes) after induced cell death.

### Statistical analysis

The statistical differences were calculated by using Student's *t* test.

## Data availability

The MS proteomics data have been deposited in the ProteomeXchange Consortium (http://proteomecentral.proteomexchange.org) via the PRIDE partner repository (23) with the data set identifiers PXD021420.
