# Supplementary material for: Phosphorylation of SMURF2 by ATM exerts a negative feedback control of DNA damage response
Source: J Biol Chem. 2021 Jan 13;295(52):18485–93. doi: 10.1074/jbc.RA120.014179 (PMC9350827; doi:10.1074/jbc.RA120.014179)
Supplement: Supplementary file 1 [file mmc1.pdf]

Phosphorylation of SMURF2 by ATM exerts a negative feedback control of DNA damage response

Liu-Ya Tang[1], Adam Thomas[1], Ming Zhou[2] and Ying E. Zhang[1,*]

Supplemental Figure

Figure S1. Camptothecin treatment induces the interaction between SMURF2 and ATM.

Supplemental Tables

Table S1. The list of SMURF2 peptides identified in control sample.

Table S2. The list of SMURF2 peptides identified in etoposide-treated sample.

Supplemental Figure S1

Camptothecin treatment induces the interaction between SMURF2 and ATM.

A. Camptothecin (CPT) treatment induces DNA damage. U2OS cells were treated with 50 µM CPT for 1 h, and γ-H2AX was detected by Western blot.

B. SMURF2 and ATM interacts in U2OS cells upon CPT treatment. U2OS cells, which transiently expressed FLAG-SMURF2 (WT or SA mutant), were treated with CPT for 1 h. Cells were analyzed by PLA using primary antibodies that recognize FLAG or ATM, respectively. Scale bar: 10 µm.

C. Quantitation of PLA signals showed that interaction between SMURF2 SA and ATM was weaker than that of SMURF2 WT.

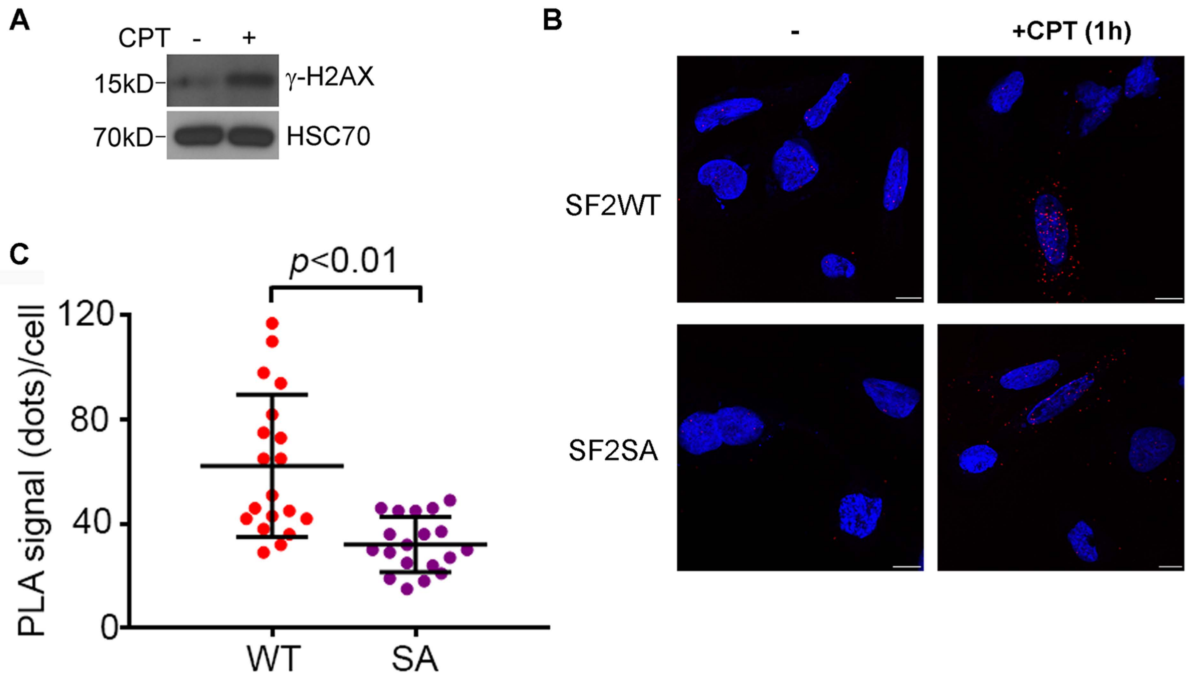

Supplemental Table S1: The list of SMURF2 peptides identified in control sample

Peptide	Annotated Sequence	Modifications	# Missed Cleavages	Charge	m/z [Da]	MH+ [Da]	Theo. MH+ [Da]	DeltaM [ppm]	XCorr
72-83	[K].SDSVTISVWNHK.[K]		0	2	686.8479	1372.68852	1372.68555	2.16	0.98
173-181	[R].IQYLNHITR.[T]		0	3	386.55206	1157.64164	1157.64257	-0.8	1.58
245-260	[R].THLHTPPDLPEGYEQR.[T]		0	3	630.64203	1889.91153	1889.91405	-1.33	2.01
261-283	[R].TTQQGQVYFLHTQTGVSTWHDPR.[V]		0	3	896.43671	2687.29557	2687.29609	-0.19	1.34
287-306	[R].DLsNINCEELGPLPPGWEIR.[N]	S3(Phospho)	0	3	778.02551	2332.06198	2332.06792	-2.55	2.31
330-340	[R].LSANLHLVLNR.[Q]		0	2	625.37128	1249.73528	1249.73753	-1.8	2.73
400-408	[R].EEIFEESYR.[Q]		0	2	601.27161	1201.53594	1201.53716	-1.02	1.46
426-439	[K].FRGEEGLDYGGVAR.[E]		1	3	509.25116	1525.73893	1525.73938	-0.3	2.52
462-488	[R].DDIYTLQINPDSAVNPEHLSYFHFVGR.[I]		0	3	1049.84399	3147.51743	3147.51704	0.12	4.43
489-510	[R].ImGmAVFHGHYIDGGFTLPFYK.[Q]	M2(Oxidation); M4(Oxidation)	0	3	845.07007	2533.19565	2533.20428	-3.41	2.81
593-603	[R].GIEAQFLALQK.[G]		0	3	406.56778	1217.68879	1217.68885	-0.05	1.39

Supplemental Table S2: The list of SMURF2 peptides identified in etoposide-treated sample

Peptide	Annotated Sequence	Modifications	# Missed Cleavages	Charge	m/z [Da]	MH+ [Da]	Theo. MH+ [Da]	DeltaM [ppm]	XCorr
32-38	[R].LPDPFAK.[IV]		0	1	787.43494	787.43494	787.43487	0.09	2.07
39-54	[K].VVVDGsGQCHSTDTVK.[N]	S6(Phospho)	0	2	856.36652	1711.72576	1711.73569	-5.8	2.85
72-83	[K].SDSVTISVWNHK.[K]		0	2	686.84796	1372.68865	1372.68555	2.25	2.39
120-129	[K].LGPNDNDTVR.[G]		0	1	1100.52979	1100.52979	1100.53308	-2.99	1.64
130-139	[R].GQIVVSLQSR.[D]		0	1	1086.62695	1086.62695	1086.62658	0.34	2.54
154-166	[R].LFDNDLPDGWEER.[R]		0	3	535.9115	1605.71994	1605.71798	1.23	2.27
154-167	[R].LFDNDLPDGWEERR.[T]		1	3	587.94446	1761.81882	1761.81909	-0.15	2.1
173-181	[R].IQYLNHITR.[T]		0	2	579.32483	1157.64238	1157.64257	-0.16	2.71
245-260	[R].THLHTPPDLPEGYEQR.[T]		0	3	630.64386	1889.91703	1889.91405	1.57	4.8
261-283	[R].TTQQGQVYFLHTQTGVSTWHDPR.[V]		0	4	672.57904	2687.29433	2687.29609	-0.65	0.87
287-306	[R].DLsNINCEELGPLPPGWEIR.[N]	S3(Phospho)	0	3	778.026	2332.06345	2332.06792	-1.92	2.43
330-340	[R].LSANLHLVLNR.[Q]		0	2	625.37183	1249.73638	1249.73753	-0.92	3
346-367	[K].DQQQQQVVSLCPDDTECLTVPR.[Y]		0	3	834.72363	2502.15635	2502.15991	-1.43	1.9
381-394	[R].QELsQQQPQAGHCR.[I]	S4(Phospho)	0	3	563.9068	1689.70584	1689.71629	-6.18	3.43
395-408	[R].IEVSREEIFEESYR.[Q]		1	3	595.96033	1785.86643	1785.86537	0.59	3.79
400-408	[R].EEIFEESYR.[Q]		0	1	1201.53723	1201.53723	1201.53716	0.06	3.17
426-439	[K].FRGEEGLDYGGVAR.[E]		1	2	763.37225	1525.73723	1525.73938	-1.41	4.53
428-439	[R].GEEGLDYGGVAR.[E]		0	2	611.78827	1222.56926	1222.56986	-0.49	2.78
462-488	[R].DDIYTLQINPDSAVNPEHLSYFHFVGR.[I]		0	4	787.63446	3147.51601	3147.51704	-0.33	6.81
489-510	[R].IMGmAVFHGHYIDGGFTLPFYK.[Q]	M4(Oxidation)	0	4	630.05853	2517.2123	2517.20937	1.16	2.87
489-510	[R].ImGmAVFHGHYIDGGFTLPFYK.[Q]	M2(Oxidation); M4(Oxidation)	0	3	845.07404	2533.20755	2533.20428	1.29	2.4
570-579	[K].SIPVNEENKK.[E]		1	2	579.31177	1157.61626	1157.61608	0.16	1.51
593-603	[R].GIEAQFLALQK.[G]		0	3	406.56763	1217.68833	1217.68885	-0.43	2.74
604-615	[K].GFNEVIPQHLLK.[T]		0	2	697.8949	1394.78252	1394.77906	2.48	2.02
616-631	[K].tFDEKELELIICGLGK.[I]	T1(Phospho)	1	2	944.45898	1887.91069	1887.91734	-3.52	2.94
659-668	[K].AVEFFDEERR.[A]		1	2	649.31262	1297.61797	1297.61714	0.64	1.7
671-680	[R].LLQFVTGSSR.[V]		0	3	369.87704	1107.61658	1107.61568	0.81	1.96
688-696	[K].ALQGAAGPR.[L]		0	1	840.46875	840.46875	840.46863	0.15	2.26
697-712	[R].LFtIHQIDACTNNLPK.[A]	T3(Phospho)	0	2	954.45361	1907.89995	1907.90851	-4.49	2.99
